# Renal Embolization-Induced Uremic Swine Model for Assessment of Next-Generation Implantable Hemodialyzers

**DOI:** 10.3390/toxins15090547

**Published:** 2023-09-04

**Authors:** Jarrett Moyer, Mark W. Wilson, Thomas A. Sorrentino, Ana Santandreu, Caressa Chen, Dean Hu, Amy Kerdok, Edward Porock, Nathan Wright, Jimmy Ly, Charles Blaha, Lynda A. Frassetto, William H. Fissell, Shant M. Vartanian, Shuvo Roy

**Affiliations:** 1 Departments of Bioengineering & Therapeutic Sciences, Surgery, Medicine, and Radiology & Biomedical Imaging, University of California, San Francisco, CA 94143, USA; jarrett.moyer@ucsf.edu (J.M.);; 2 Silicon Kidney, San Ramon, CA 94583, USA; 3 Outset Medical, San Jose, CA 95134, USA; 4 Division of Nephrology & Hypertension, Vanderbilt University Medical Center, Nashville, TN 37232, USA

**Keywords:** end stage renal disease, acute kidney injury, chronic kidney disease, dialysis, hemodialysis, artificial organs

## Abstract

Reliable models of renal failure in large animals are critical to the successful translation of the next generation of renal replacement therapies (RRT) into humans. While models exist for the induction of renal failure, none are optimized for the implantation of devices to the retroperitoneal vasculature. We successfully piloted an embolization-to-implantation protocol enabling the first implant of a silicon nanopore membrane hemodialyzer (SNMHD) in a swine renal failure model. Renal arterial embolization is a non-invasive approach to near-total nephrectomy that preserves retroperitoneal anatomy for device implants. Silicon nanopore membranes (SNM) are efficient blood-compatible membranes that enable novel approaches to RRT. Yucatan minipigs underwent staged bilateral renal arterial embolization to induce renal failure, managed by intermittent hemodialysis. A small-scale arteriovenous SNMHD prototype was implanted into the retroperitoneum. Dialysate catheters were tunneled externally for connection to a dialysate recirculation pump. SNMHD clearance was determined by intermittent sampling of recirculating dialysate. Creatinine and urea clearance through the SNMHD were 76–105 mL/min/m^2^ and 140–165 mL/min/m^2^, respectively, without albumin leakage. Normalized creatinine and urea clearance measured in the SNMHD may translate to a fully implantable clinical-scale device. This pilot study establishes a path toward therapeutic testing of the clinical-scale SNMHD and other implantable RRT devices.

## 1. Introduction

End-stage renal disease (ESRD) is a global epidemic and affects more than 800,000 patients in the United States alone. Currently there is limited availability of organs for kidney transplant, the gold-standard treatment for ESRD, with almost 100,000 patients on the waiting list for a transplant but around 25,000 operations performed each year [1]. Therefore, the vast majority of patients must rely on dialysis for renal replacement therapy, and nearly 90% undergo in-center hemodialysis (HD) as their primary dialytic modality [2]. Despite the ability of HD to successfully perform solute exchange and clear uremic toxins, patients still suffer other effects of ESRD including cognitive disorders and anemia, as well as a significant decrease in ability to maintain employment [3,4,5,6]. In addition, nocturnal home HD is becoming more prevalent and is more physiologically optimal than standard in-center HD but comes with higher risks of technical complications [2,7]. The low rate of kidney transplants and limitations of traditional hemodialysis reveal a clear need to develop novel technologies for the next generation of replacement therapies, including (bio)artificial kidneys and safer methods of home HD.

Several groups are developing portable or implantable artificial kidneys and hemodialyzer technologies [8,9,10,11,12,13,14,15,16,17]. Notable early attempts at developing a bioartificial kidney were pioneered by Aebischer and colleagues, who successfully cultured renal cell lines on semipermeable hollow fiber membranes [17]. The group led by Humes next developed the “Renal Assist Device” (RAD) wherein a standard hollow fiber hemofilter was placed in series with a second hollow fiber membrane containing human renal cells. This device was successfully tested in animal studies and a combined Phase I/II study in humans, leading to a randomized Phase II trial against standard continuous RRT in critically ill patients with renal failure. Use of the RAD resulted in an approximately 50% reduction in mortality compared to standard CRRT, but further studies were abandoned due to problems with manufacturing and study design [18]. More recently, a number of wearable renal replacement systems have been developed, including the WEAKID and automated wearable artificial kidney (AWAK) systems for peritoneal dialysis and the wearable artificial kidney (WAK) system for hemodialysis [8,19,20]. These devices showcase innovation in the miniaturization and portability of dialysis equipment through advances in dialysate regeneration, novel dialysate compounds, and solute exchange methods. Unfortunately, however, none of these devices have been approved for clinical use in humans outside of a trial.

An important step in device translation to human studies is demonstrating robust safety and efficacy data in a large animal model. To test the functional characteristics of an implantable renal replacement therapy, there is a need for an animal model with compromised renal function. An ideal model should replicate human renal physiology and anatomy, induce stable renal failure while remaining compatible with standard hemodialysis techniques, and preserve surgical anatomy for implantation of experimental RRT devices. Animal models with some of these characteristics have been established in swine, sheep, and goats [21,22,23,24]. Specifically in swine, several models have been developed to induce acute and chronic renal failure via surgery, ischemia-reperfusion (IR) injury, sepsis, nephrotoxic drugs, and combinations these modalities. Laparoscopic 5/6th nephrectomy has been shown to be safe and more effective at rapidly inducing chronic renal failure compared to 2/3rd or 3/4th nephrectomy [25]. IR injury models for induction of renal failure are detailed in a variety of reports; however, the methods are heterogeneous. Open, laparoscopic, and endovascular techniques with varying durations of either arterial or arteriovenous occlusion, with or without contralateral nephrectomy, have been described, leading to a wide severity and duration of renal dysfunction [26,27,28]. Moreover, there is a very narrow window of duration of ischemic injury that leads to chronic renal injury; a shorter time allows for renal recovery, and a longer time creates significant risk for animal mortality [29]. Sepsis-induced renal injury, while effective at inducing renal injury, creates multi-organ system dysfunction that would compromise downstream evaluation of novel RRT [30]. Finally, drug-induced renal injury has been combined with surgical nephrectomy to induce acute-on-chronic renal injury to study reno-protective drug therapies as well at RRT [22,31,32]. Swine subtotal endovascular nephrectomy, originally described by Misra and colleagues, effectively and reproducibly induces chronic renal failure and leaves retroperitoneal anatomy intact for subsequent device implants [24]. To date, however, this model has not been used to study implantable hemodialysis-based RRT.

A key aspect of novel RRT is engineering high-mass transport, selective permeability, and biocompatible membranes to replace traditional hollow-fiber hemodialyzer filters. Silicon nanopore membranes (SNM) are a new class of such membranes manufactured via semiconductor fabrication methods, allowing for precise control of pore size. Our group has utilized these techniques to manufacture membranes with 5–10 nm pores that enable diffusive mass transfer of small molecules while serving as a physical immunobarrier to protect encapsulated cells from the host immune system [33,34,35,36,37]. Other silicon-based platforms have been developed to achieve similar solute filtration characteristics [38,39]. These favorable mass transfer characteristics are coupled with surface modification chemistries to enhance the anti-protein fouling capabilities and reduce membrane thrombogenicity [40,41,42]. We have previously demonstrated proof-of-concept of implantable SNM hemofilters designed to simulate glomerular filtration in both healthy dog and swine models [12,43].

In this article, we present a pilot embolization-to-implantation protocol that can serve as a platform for testing the next generation of RRT devices. We developed a staged, endovascular nephrectomy that leads to stable renal failure that can be managed with HD and does not disturb the retroperitoneal structures critical for device implantation. To demonstrate the utility of protocol for surgically implanted devices, we then show proof-of-concept using a small-scale implantable SNM hemodialyzer (SNMHD) capable of clearing uremic toxins without albumin loss.

## 2. Results

### 2.1. Staged Bilateral Renal Artery Embolization Induces a Stable Renal Failure State

**Figure 1:**
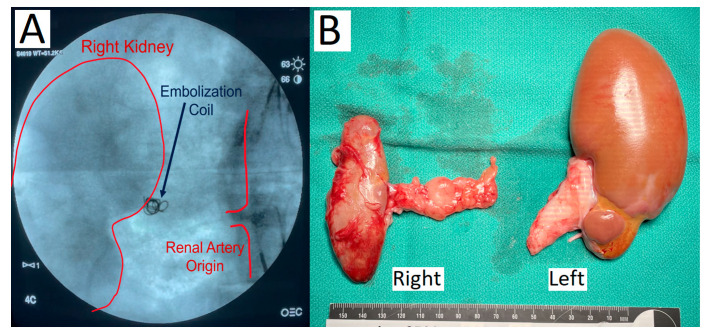
(**A**) Fluoroscopic angiography demonstrating PVA particle and coil embolization of the right renal artery resulting in contrast stasis. (**B**) Necropsy of porcine kidneys shows atrophy and fibrosis of the entire right kidney and the inferior pole of the left kidney with hypertrophy of the superior pole.

**Figure 2:**
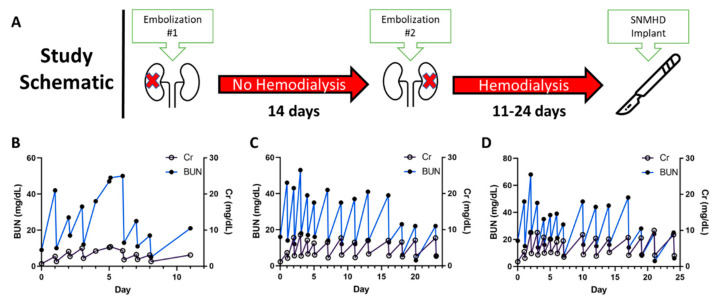
(**A**) Staged bilateral renal artery embolization was performed on day 0 and day 14 with hemodialysis initiation after the second embolization. (**B**–**D**) Uremia was managed in all three animals with intermittent hemodialysis every 1–3 days for 11–24 days. Pre- and post-HD serum BUN and Cr values are shown individually for each animal. The SNMHD was ultimately implanted in the third animal at day 24 (**D**).

Three Yucatan minipigs underwent staged bilateral subtotal renal artery embolization. The approach of a two-stage complete right renal artery embolization followed by selective embolization of the inferior renal pole of the left kidney led to a state of stable renal failure managed by hemodialysis. Initial embolization of the main right renal artery with PVA particles and microcoils leading to stasis of contrast flow into the kidney is shown in Figure 1A. The animals recovered for 14 days, then underwent the second stage partial left renal embolization, which induced uremia (Figure 2A). After the second embolization, the baseline blood urea nitrogen (BUN) range of 9–19 mg/dL increased to a maximum of 49–68 mg/dL and baseline creatinine (Cr) range of 0.7–1.3 mg/dL increased to a maximum of 5.2–10.0 mg/dL. All animals reached a steady-state of renal failure that was managed with hemodialysis every 1–3 days using a Tablo dialysis system (Figure 2B–D). Aside from uremia and mild anemia, the animals remained healthy and without mobility issues while undergoing hemodialysis. Necropsy demonstrated atrophy and fibrosis of the entire right kidney and inferior pole of the left kidney with reciprocal hypertrophy of the superior pole of the left kidney (Figure 1B).

### 2.2. SNMHD Retroperitoneal Implantation and Patency

**Figure 3:**
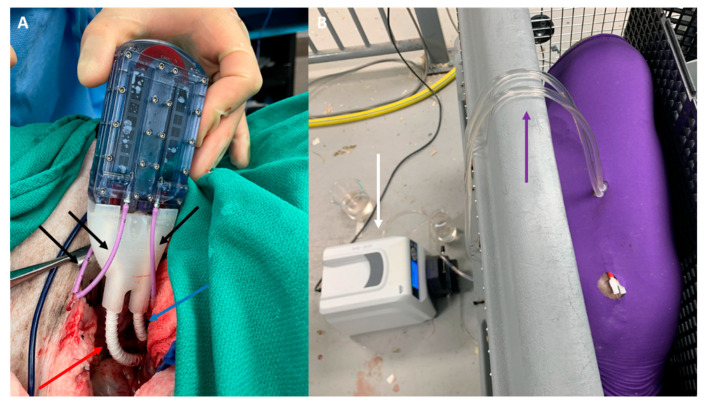
(**A**) SNMHD implanted in the retroperitoneum of a pig. Blood flows in and out via 6 mm PTFE grafts anastomosed to the external iliac artery (red arrow) and vein (blue arrow), respectively. Dialysate flows out via polyurethane catheters (black arrows). (**B**) Pig undergoing recirculation dialysis via dialysis catheters tunneled out of the skin (purple arrow), connected to a circulation pump (white arrow).

The SNMHD was implanted in one animal in the retroperitoneum, with vascular connections to the right iliac artery and vein. Dialysis catheters were externalized via the flank to allow circulation of normal saline used in place of dialysate solution into device dialysate manifolds (Figure 3A). There were no immediate technical complications, and the animal had no device- or procedure-related complications for the duration of the 7-day study. The blood flow path in the device remained patent through post-procedure day 7 without system anticoagulation therapy. On explant, no macroscopic thrombus was observed on the silicon membrane.

### 2.3. SNMHD Effectively Clears Potassium, Creatinine, BUN While Preventing Albumin Loss

**Figure 4:**
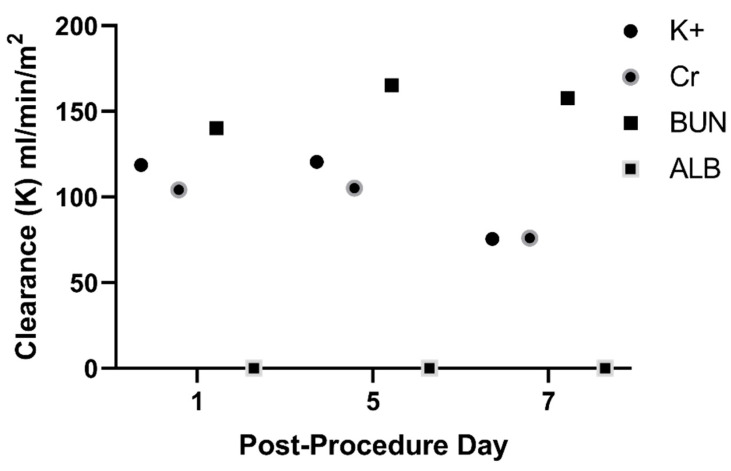
SNMHD effectively clears potassium (K+), creatinine (Cr), and urea (BUN) on post-procedure days 1, 5, and 7. No albumin was detected in the dialysate at any of the three timepoints.

Recirculation dialysis with the SNMHD was performed for 2 h on post-implant days 1, 5, and 7 prior to standard hemodialysis (Figure 3B). Serum electrolytes, albumin, BUN, and Cr values were measured at time 0, and recirculated dialysate was sampled for the same measurements at times 0, 60, and 120 min. Solute clearance normalized to membrane surface area for Cr was 76–105 mL/min/m^2^, for BUN was 140–165 mL/min/m^2^, and for potassium (K+) was 75–120 mL/min/m^2^ after 120 min of dialysis with the SNMHD (Figure 4). No albumin was detected in the dialysate at any time point, demonstrating that the SNM barrier remained intact.

## 3. Discussion

In this report, we demonstrate the successful pilot of an embolization-to-implantation protocol enabling the first implant of a silicon nanopore membrane hemodialyzer in a swine model of renal failure. Staged endovascular nephrectomy resulted in stable renal failure managed with standard hemodialysis while preserving the critical anatomy for SNMHD implantation. A small-scale SNMHD was successfully implanted, remained patent for the entire 7-day study without systemic anticoagulation, and cleared uremic toxins and solutes without leakage of albumin. Amongst large animal species for studying renal failure and novel renal replacement therapies for translation to humans, swine offer several attractive attributes. Porcine kidneys have a similar lobed, pyramidal architecture to human kidneys, are relatively comparable in size, and have analogous vascular anatomy and physiology [44,45,46]. Moreover, pigs have comparable values of serum electrolytes, BUN, and creatinine to humans [47]. While the concept of endovascular nephrectomy for studying renal failure in swine was initially reported nearly two decades ago, other methods of inducing renal failure, including open or laparoscopic surgery and drug-induced kidney injury, predominate [24,30]. Misra and colleagues found that single-stage bilateral endovascular nephrectomy can produce mobility-limiting pain and an overwhelming inflammatory response that is life-threatening for the animal [24]. Recently, a similar endovascular nephrectomy protocol has been used to induce uremia in swine for the study of novel peritoneal dialysis therapies [22]. The same group has also developed an acute-on-chronic goat model of kidney injury using a combination of endovascular nephrectomy and gentamicin-induced kidney injury with animals surviving for 10 months; a procedure which may be advantageous for studying chronic dialysis therapies [23]. In our protocol, we found that a staged approach results in a state of renal failure manageable with initial daily hemodialysis for one week, followed by hemodialysis every 2–3 days, and allows the animals to stay healthy for at least one month (see Table 1 for detailed comparison of the surgical approaches). Importantly, this approach preserves the retroperitoneal anatomic planes and vasculature, facilitating straightforward exposure of the iliac vasculature for anastomosis of the SNMHD or other implantable, blood-based renal replacement technologies.

Compared to the approaches described by de Vries [22] and Misra [24], our approach offers at least two distinct advantages. First, both groups reported fatal or euthanasia-requiring complications associated with their single-stage embolization procedures. This is consistent with our group’s attempts at single-stage embolization, performed prior to this study, that led to hindlimb paralysis and uncontrollable electrolyte abnormalities leading to death. Second, our approach with a two-stage subtotal embolization in Yucatan minipigs leads to a state of chronic renal failure that is more profound than previous reports. The protocol described by Misra et al. led to kidney injury not requiring dialysis, and the protocol of de Vries et al. utilized the addition of gentamicin to induce a degree of renal failure needing dialysis. Our protocol leads to stable renal failure requiring dialysis after the second embolization, making it ideal for studying implantable renal replacement therapies.

The SNMHD implanted in this study builds on a significant body of work by our group and others. Previously, we have successfully implanted both smaller and similar scale silicon nanopore membrane devices via anastomoses to the carotid artery and external jugular vein in healthy swine [12,42]. Silicon nitride nanoporous-based hemodialyzers have been tested with extracorporeal circulation in rats with adenine-induced renal dysfunction and shown to clear uremic toxins without albumin spillage [39]. The protocol described in this study offers a platform for scaling up to devices with clinically therapeutic dialysis capacity.

In contrast to traditional hollow-fiber dialysis membrane, which have wide variation in pore size and are thrombogenic even with anticoagulation, implantable SNMHD are engineered with antifouling surface coatings and laminar blood flow path to decrease protein aggregation and reduce thrombogenicity. Previously, SNM have been coated with polyethylene glycol (PEG) and polysulfobetaine methacrylate (pSBMA) thin films; however, PEG is prone to degradation under biologic conditions and pSBMA synthesis and deposition is complex [34,42,48]. Alumina thin-film coating via atomic layer deposition (ALD) was utilized due to its durability, anti-fouling, and anti-thrombogenic properties [40,41]. Device patency and clearance of electrolytes, BUN, and Cr over the 7-day study period with only oral dual antiplatelet medication is reflective of the hemocompatibility of the SNM surface coating. Of note, while the clearance of Cr and K^+^ decreased over the 7-day study, the clearance of urea remained stable. In general, protein fouling of the membrane leads to decreased solute clearance, so it is unclear why changes in solute clearance were not similar for all solutes, necessitating further investigation. Engineering of robust, anti-thrombogenic thin-film surfaces is an active area of research and crucial to the success of any artificial, implantable renal replacement therapy.

This study has a few clear limitations. First, only three animals underwent embolization and only one device implant is described in this study. Further studies are underway with a larger-scale SNMHD to validate and optimize the techniques described here. Next, the device was implanted for only 7 days. Although the flow path remained patent and the device cleared small molecule solute consistently without therapeutic anticoagulation, a clinically practical implantable hemodialyzer will need to remain patent for months to years. Future studies will study both longevity of our current devices and modifications required to extend duration of device patency. A combination of a fluid dynamics-optimized flow path and continued advances in anti-fouling surface coatings research is crucial for the success of a long-term implanted device. Finally, as with any externalized catheters, there is an infection risk that can lead to significant complications. Advances in regenerative dialysate and implantable renal cell bioreactors will be necessary to realize the goal of a fully contained, implantable hemodialyzer or bioartificial kidney [14].

## 4. Conclusions

We report an embolization-to-implantation protocol for inducing renal failure via staged endovascular nephrectomy, while preserving retroperitoneal structures critical for device implantation in a swine model. We demonstrated successful retroperitoneal implantation of a small-scale SNMHD. The device remained patent for 7 days and cleared small molecule solutes. This is an important proof-of-concept study in the progression towards therapeutic scale implantable RRT devices.

## 5. Materials and Methods

### 5.1. Silicon Nanopore Membrane Fabrication

Silicon nanopore membranes (SNM) were fabricated as previously described [33,34]. Uniformly distributed nanopores enable high mass transport of nutrients while blocking passage of immune cells, blood, antibodies, and complement. In brief, a polysilicon coating was first deposited on 400 μm-thick silicon wafers via low-pressure chemical vapor deposition. The polysilicon film was then patterned and oxidized to conformally grow a 10 nm-thick layer of silicon dioxide (SiO_2_). An additional polysilicon layer was then deposited and planarized to create 10 nm wide veins of silicon dioxide (SiO_2_) between the polysilicon layers. The other side of the wafer was subsequently patterned with deep reactive ion etching to create a thin polysilicon membrane. The 10 nm pores were finally created by etching away the SiO_2_. The wafers were then diced into 10 mm × 65 mm membrane chips with a total porous membrane area of 2.16 cm^2^. Finally, SNM chips were coated with a 5 nm layer of alumina (Al_2_O_3_) via atomic layer deposition (ALD) using a Cambridge Fiji F2000 System (Integrated Surface Technologies, Menlo Park, CA, USA), which acts as a smoothing and anti-thrombogenic surface coating [40,41,49]. See Supplemental Figure S1 for additional details of the SNM structure [50].

### 5.2. Construction of Silicon Nanopore Membrane Hemodialyzer

**Figure 5:**
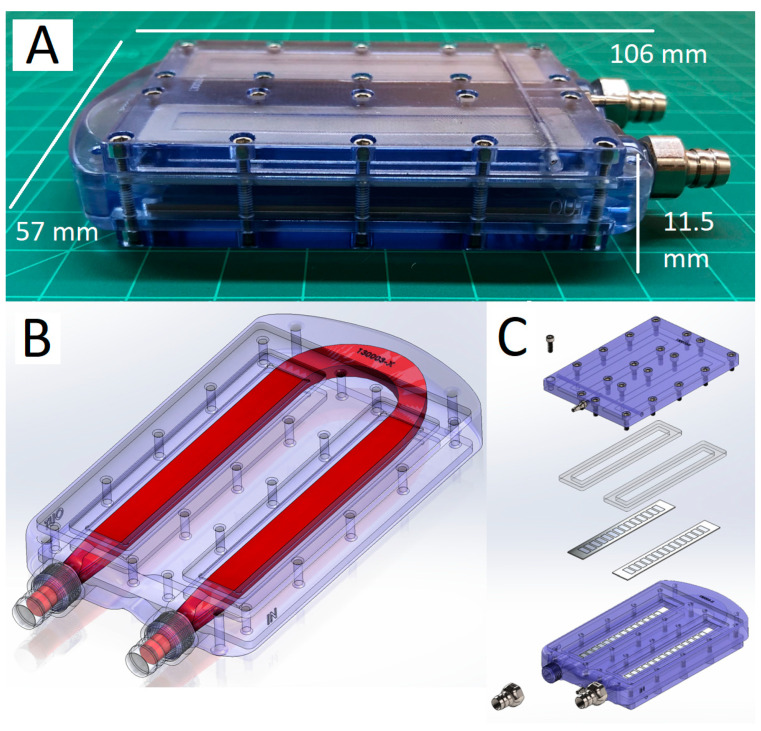
SNMHD schematic and assembly. (**A**) Assembled SNMHD consisting of a polycarbonate blood flow housing, SNM, and stainless-steel graft and dialysate connectors (106 mm L × 57 mm W × 11.5 mm H). (**B**) CAD image demonstrating blood flow path geometry. (**C**) An exploded view of the SNMHD, showing that the blood path is bordered by parallel SNM and held in place by compressive forces provided by the polycarbonate dialysate manifold and silicone gaskets.

A custom SNMHD was fabricated from polycarbonate and containing SNM to facilitate intracorporeal dialysis. In brief, we created a modified version of our single-channel, two-SNM chip flow chamber with the addition of dialysate manifold outflow chambers to enable external dialysate recirculation [42]. Designs were generated using SolidWorks (Dassault Systèmes, Waltham, MA, USA) and computational fluid dynamic modeling was used to optimize the blood flow path with Ansys Fluent (Ansys, Inc., Canonsburg, PA, USA). Polycarbonate housings were fabricated via CNC machining (Prototek, San Jose, CA, USA). Graft connectors were milled from 316L stainless steel (Prototek), and silicone gaskets were laser cut from USP VI grade silicone. The device was assembled as shown in Figure 5.

### 5.3. Swine Renal Artery Embolization and Hemodialysis

Swine renal arterial embolization, device implantation, and dialysis were carried out under an IACUC-approved protocol at a contract research organization facility (Labcorp, San Carlos, CA, USA). A healthy juvenile Yucatan minipig underwent staged bilateral renal artery embolization using a modification of a previously published protocol [24]. The animal was started on aspirin 81 mg daily and clopidogrel 75 mg daily for three days prior to initial embolization. Trimethoprim/sulfamethoxazole 15 mg/kg was given daily as antibiotic prophylaxis against central venous catheter infection for the duration of the study. On the day of initial embolization, a double lumen central venous HD catheter (Palindrome 14.5Fr Chronic Dialysis Catheter, Insertion Length 33 cm, Covidien-Medtronic, Minneapolis, MN, USA) was placed via surgical cutdown into the external jugular vein to enable subsequent HD and blood draws. The right renal artery was completely embolized using a combination of spherical polyvinyl alcohol particles (Contour Embolization Particles, 355–500 micron and 500–710 micron, Boston Scientific Corporation, Marlborough, MA, USA) and embolic microcoils (ev3 Concerto Detachable Coil System, 0.29 mm × 3 mm × 4 cm, Micro Therapeutics, Inc., Irvine, CA, USA). The embolic particles were introduced through a 5 Fr C2 Cobra angiographic catheter (Cook Medical, Bloomington, IN, USA) with a coaxial 2.8 Fr Progreat microcatheter (Terumo Corporation, Shibuya City, Tokyo, Japan), inserted into the renal artery via a right common femoral artery sheath (Arrow 6Fr Sheath Introducer, Teleflex, Morrisville, NC, USA). Embolization was confirmed via digital subtraction angiography with nonionic contrast media (Omnipaque 300 (Iohexol), GE Healthcare, Inc., Chicago, IL, USA).

The animal was allowed to recover for 14 days, after which partial embolization of the contralateral kidney was performed under general anesthesia as described above, with embolization spheres injected into the inferior pole renal artery branch after selective catheterization. Following the second embolization, the animal underwent daily intermittent hemodialysis on the Tablo Hemodialysis System (Outset Medical, San Jose, CA, USA) via the central venous catheter for seven days, followed by thrice-weekly HD on days 8+. Hemodialysis treatments aimed to maintain euvolemia by titrating ultrafiltration to animal weight on treatment days. The hemodialysis prescription was tailored to blood chemistry analysis (Idexx Catalyst Dx Chemistry Analyzer, Idexx Laboratories, Inc. Westbrook, MN, USA) on the day of treatment to maintain acid–base balance and achieve post-treatment serum potassium concentration less than 4.0 mEq/L. Treatment duration was titrated to achieve a urea reduction ratio greater than 60% during daily treatment periods and greater than 70% during thrice-weekly periods. Sessions typically lasted between 90–180 min.

### 5.4. SNMHD Implantation Procedure

Device implantation was performed 25 days after induction of uremia, allowing time for the animal to recover from the second embolization and enter a state of stable renal failure. The device was assembled as above, sterilized with 70% EtOH for 30 min, and purged with sterile water. Prior to implantation, the device was primed with sterile 0.9% NaCl solution. After induction of anesthesia, a curvilinear incision was made in the right lower quadrant and extended through the abdominal wall musculature. The peritoneum was swept superiomedially, and dissection was carried down to expose the right iliac artery and vein. The vessels were isolated, 100 U/kg of heparin was given intravenously, and the vessels were clamped. Grafts of 6 mm ringed polytetrafluoroethylene (PTFE) (Gore Medical, Santa Clara, CA, USA) were sewn to the artery and vein with 6–0 Prolene suture (Ethicon, Raritan, NJ, USA) in an end-to-side fashion. The distal ends of the PTFE grafts were covered with a custom-molded DragonSkin^TM^ silicone protector (10 Slow, Smooth-On Inc., Macungie, PA, USA) to prevent graft kinks. The arterial and venous grafts were connected to the metal barbs on the inflow and outflow sides of the flow chamber, respectively. Sterile polyurethane tubing (PowerHickman 8.0 Fr Single Lumen Catheter, BD, Franklin Lakes, NJ, USA) was connected to the dialysate outflow barbs and tunneled externally through the subcutaneous tissues, then the incision was closed in layers.

### 5.5. Post-Procedure Animal and Device Dialysis and Solute Measurement

**Figure 6:**
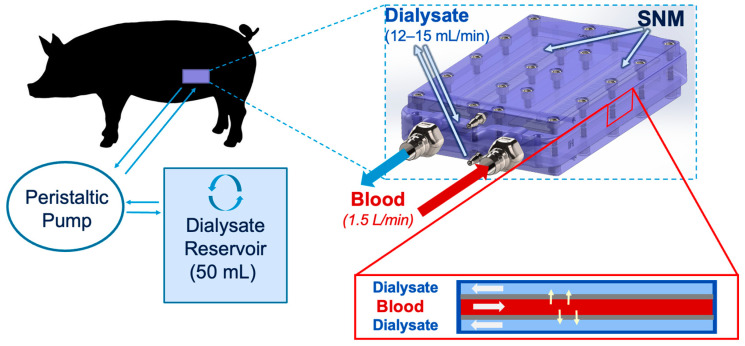
SNMHD dialysate recirculation schematic. Dialysate is recirculated from a reservoir and sampled over time. Solute exchange occurs through the SNM interface between the blood and dialysate chambers. Changes in solute concentration allow measurement of normalized SNMHD clearance.

The animal underwent dialysis with recirculated dialysate with the implanted SNMHD on post-procedure days 1, 5, and 7, prior to standard HD for 2 h. Due to the sub-clinical membrane area of the SNMHD, and our intent to initially evaluate clearance potential for a future clinically-sized device, a closed dialysate recirculation system was used to measure solute clearance over time (Figure 6). To garner these insights, a 0.9% NaCl solution (Aqualite System, Hospira, Inc., Lake Forest, IL, USA) was used to create a 50 mL “dialysate” reservoir and to prime IV tubing, which then attached to the previously externalized polyurethane tubing connected to the dialysate manifold of the SNMHD. NaCl solution was chosen as it is a readily available solution that does not contain our solutes of interest, namely potassium, urea, and creatinine. NaCl solution was continuously circulated through the SNMHD using an infusion pump (Heska Corp, Loveland, CO, USA) at a rate of 15 mL/min in order to maintain a transmembrane pressure of 0 mmHg and prevent ultrafiltration. Blood samples were collected from the pig at initiation of recirculation dialysis for measurement of serum electrolytes, BUN, Cr, and albumin. Recirculated dialysate was collected hourly for the same measurements. Concentrations of Cr, BUN, and albumin in serum and from the dialysate reservoir were measured using an Avida 1800 Chemistry System (Siemens Medical, Erlangen, Germany) at Zuckerberg San Francisco General Hospital and Trauma Center (San Francisco, CA, USA). The coefficient of variation machine error for the Avida system was <4% for all assays. Changes in solute concentration allowed measurement of normalized SNMHD clearance. Clearance, *K*, was calculated according to:

$$K=\left(C_{d}\times V\right)/\left(C_{p}\times t\times A_{m}\right)$$

where *C_d_* is dialysate concentration, *C_p_* is plasma concentration, *V* is dialysate volume, *t* is time, and *A_m_* is membrane area. We assumed *C_p_* to be constant in the setting of sub-clinical SNMHD devices based on prior in vitro and in vivo experiments [33].

### 5.6. Explant and Retrieval of the SNM Hemodialyzer

Following completion of the study protocol, the animal was anesthetized for device retrieval. Following induction of general anesthesia, the SNMHD was exposed surgically and recovered. The device was gently flushed with 1 L of normal saline to prevent subsequent gross thrombosis of static blood and placed into a normal saline bath for transport from the animal facility. After device disassembly, the SNM were grossly examined for defects and evidence of thrombi. The animal was euthanized according to AVMA Guidelines for Euthanasia of Animals [51].

## Figures and Tables

**Table 1:** Comparison of details of surgical techniques for swine renal artery embolization and device implantation (current work, de Vries et al. [22], and Misra et al. [24]).

	Current Work	de Vries et al.	Misra et al.
Animal	Female Yucatan minipig (age: 4–14 months, weight: 43–84 kg)	Female Yorkshire pig (age: 4 months, weight ~40 kg)	Male domestic pig (age: 7–8 months old, weight: 35 ± 1 kg)
Premedication	Antiplatelets: 3 days prior to device implant: Aspirin 81 mg daily, clopidogrel 75 mg daily Antibiotics: Trimethoprim/sulfamethoxazole 15 mg/kg daily starting day of device implant and continued for duration of study	Antibiotics: Amoxicillin/clavulanic acid 10 mg/kg × 1 pre-operatively, then twice daily after PD catheter insertion	None described
Anesthesia and Post-Operative Analgesia	Premedication: Telazol + atropine General anesthetic: Isoflurane +/− ketamine Intraoperative analgesia: Butorphanol + buprenorphine Postoperative analgesia: Buprenorphine IM/IV + NSAID per veterinary discretion	Premedication: Ketamine + atropine General anesthetic: Propofol Intraoperative analgesia: Remifentanil Postoperative analgesia: Meloxicam ×1 day, buphrenophine patch ×6 days	General anesthesia for embolization and additional procedures. Other details not provided.
Embolization Procedure	Dual staged embolization: Complete right renal artery embolization with PVA particles and microcoils; 14-day recovery; partial left inferior pole renal artery embolization	Single stage sub-total renal artery embolization (one kidney completely embolized, other partially) with PVA particles or microspheres	Single stage sub-total renal artery embolization (one kidney completely embolized, the other partially) with PVA particles and microcoils
Surgical Technique for Device Implantation	Implantation of hemodialyzer into left retroperitoneum with vascular connections to iliac vessels and dialysis catheters tunneled externally	Insertion of peritoneal dialysis catheter inserted via standard subcutaneous tunneling method	No device implant
Grade of Kidney Dysfunction	Peak creatinine: 8.0 ± 2.4 mg/dL Peak BUN: 57.0 ± 9.6 mg/dL	Peak creatinine: 10.5 ± 5.3 mg/dL Peak BUN: 46.7 ± 14.8 mg/dL	Peak creatinine: 5.7 ± 2.8 Peak BUN: 56.5 ± 31.7 mg/dL
Complications	None	Pulmonary hemorrhage in two animals after embolization with PVA; no further complications after change to embolization with microspheres	Five animals died in pilot group: one from hyperkalemia, one euthanized for hindlimb paralysis, and three following general anesthesia for blood draws. Three animals died in the definitive group: one immediately after embolization, and two euthanized for hindlimb paralysis.
Maximum Duration of Study	31 days	285 days	84 days

## Data Availability

The data presented in this study are available on request from the corresponding author.

## Supplementary Materials

The following supporting information can be downloaded at: https://www.mdpi.com/article/10.3390/toxins15090547/s1, Figure S1: Conceptual diagram of flat (a) and ribbed silicon nanopore membrane (b). 3D rendering of orthogonal backside rib network with blood and ultrafiltrate sides shown (c). Scanning electron microscopy image of SNM (d).
