# Supplementary material for: Renal Embolization-Induced Uremic Swine Model for Assessment of Next-Generation Implantable Hemodialyzers
Source: Toxins (Basel). 2023 Sep 4;15(9):547. doi: 10.3390/toxins15090547 (PMC10536310; doi:10.3390/toxins15090547)
Supplement: Supplementary file 1 [file toxins-15-00547-s001.zip › toxins-2327687-supplementary.pdf]

toxins

Supplementary Materials: Renal Embolization-Induced Uremic Swine Model for Assessment of Next-Generation Implantable Hemodialyzers

Jarrett Moyer, Mark W. Wilson, Thomas A. Sorrentino, Ana Santandreu, Caressa Chen, Dean Hu, Amy Kerdok, Edward Porock, Nathan Wright, Jimmy Ly, Charles Blaha, Lynda A. Frassetto, William H. Fissell, Shant M. Vartanian and Shuvo Roy

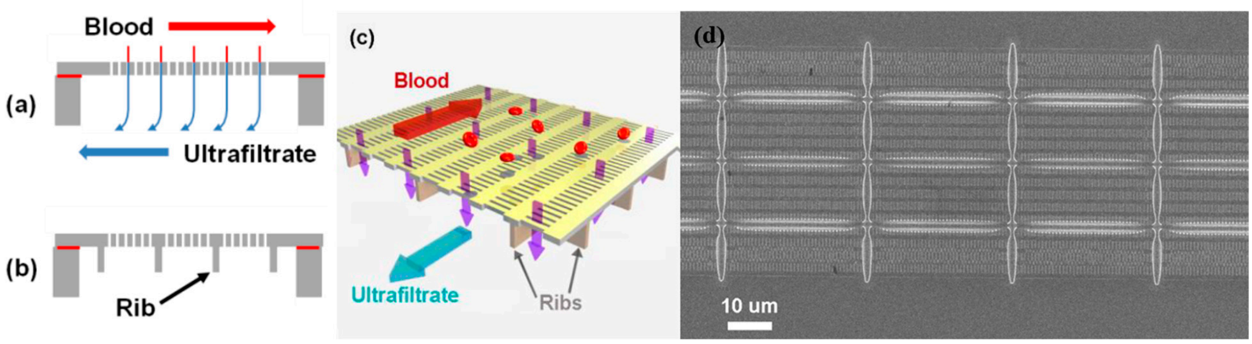

Figure S1. Conceptual diagram of flat (a) and ribbed silicon nanopore membrane (b). 3D rendering of orthogonal backside rib network with blood and ultrafiltrate sides shown (c). Scanning electron microscopy image of SNM (d).
